# Flotation—A New Method to Circumvent PCR Inhibitors in the Diagnosis of *Lawsonia intracellularis*

**DOI:** 10.1155/2009/410945

**Published:** 2009-06-17

**Authors:** Magdalena Jacobson, Börje Norling, Anders Gunnarson, Anna Aspan

**Affiliations:** ^1^Department of Clinical Sciences, Faculty of Veterinary Medicine and Animal Husbandry, P. O. Box 7018, 750 07 Uppsala, Sweden; ^2^Quintessence Research AB (QRAB), 747 91 Alunda, Sweden; ^3^National Veterinary Institute, 751 89 Uppsala, Sweden

## Abstract

The obligate intracellular bacterium *Lawsonia intracellularis* causes enteritis and poor growth in weaned pigs. Cultivation is difficult and diagnosis *ante mortem* is mainly based on techniques such as polymerase chain reaction. However, false negative results caused by the presence of PCR-inhibitory factors constitute a problem. This study aimed to develop and evaluate a new technique, flotation, to separate *L. intracellularis* from inhibitors in faeces prior to PCR. The technique was evaluated by comparison to two previously evaluated and commonly used methods, preparation by boiling lysate combined with nested PCR and preparation by a commercial kit combined with conventional PCR. Continuous density centrifugation of faecal samples containing *L. intracellularis* suggested the buoyant density of the microbe to be between 1.064 and 1.077 g/mL. Several flotation setups were tested to achieve optimal separation of the microbe from inhibitors and faecal particles. The finally selected setup floated whole *L. intracellularis* from the application site at the bottom to the upper part of the gradient while inhibitory components mainly remained in the bottom. PCR was performed directly on material recovered from the upper interphase. The method was evaluated on 116 clinical samples. As compared to sample preparation by boiling combined with nested PCR, fewer samples were inhibited but also fewer positives were identified. In comparison to preparation by a commercial kit combined with conventional PCR, presently used for routine diagnosis, similar results were obtained. However, the new method was comparably faster to perform. The new method, based on flotation of *Lawsonia intracellularis* combined with conventional PCR, was well suited for routine diagnosis.

## 1. Introduction

Enteric diseases constitute a major problem in growing pigs and have a large economic impact on pig production. However, clinical signs such as diarrhoea and poor growth may be caused by a wide range of microorganisms such as parasites, bacteria, and viruses. Hence, to implement adequate treatment and prophylactic strategies it is important to identify the causative organism and the necessity of a reliable diagnosis is obvious. The diagnostic methods required should be sensitive and specific and ideally also cheap, fast, robust, readily available, and easy to handle [1]. All these requirements can rarely be fulfilled and there is an ongoing need for further improvement of already existing methods and the development of new techniques.

The bacterium *Lawsonia intracellularis* is a major cause of enteritis in weaned pigs and is demonstrated in up to 94% of the herds [2]. Several diagnostic methods have been developed, each comprising specific advantages and disadvantages. Necropsy is used to establish a relationship between clinical signs and the presence of representative lesions but to specifically identify the causative agent; other techniques must be added [3, 4]. Further, serological methods are cheap, fast, and easy to handle and are used to scan large numbers of samples. However, a causal relationship will not be established and false reactions may be difficult to interpret [5–7]. Identification of specific DNA by polymerase chain reaction (PCR) and immunohistochemistry (IHC) on tissue samples have been suggested as “gold standard” [8, 9]. Antemortem, culture is used as gold standard for many bacteria but is presently not applicable in the routine diagnosis of the obligate intracellular *L. intracellularis* and presently, PCR or immunperoxidase staining of faecal smears is the only option available [6, 10]. Today, most laboratories utilize PCR that is fast, sensitive, and specific in the demonstration of bacterial DNA in tissue or faeces. However, when applied on complex biological samples such as faeces, false negative results constitute a problem [5, 6, 11–13]. In addition, the technique does not distinguish between dead or live bacteria and the quality and yield of DNA may depend on the target, the sample composition, and the method used for purification of DNA [1, 5]. 

Recently, a new method to separate particles and bacteria based on their buoyant density has been described. The method was successfully applied to separate *Yersinia enterocolitica* and *Campylobacter* species from food particles and inhibitors [14, 15] and was stated to enable the separation between live and dead bacteria as well as free bacterial DNA. The method has only to a limited extent been applied on faecal samples [1]. 

The purpose of this study was to develop and evaluate a new method in the diagnosis of *L. intracellularis*, based on the separation of *L. intracellularis* cells from faecal samples by flotation prior to PCR.

## 2. Material and Methods

### 2.1. Samples

Faeces were collected from Swedish commercial pigs with or without diarrhoea. For comparison, 0.1 g of all samples was also lysed by boiling and analysed for *L. intracellularis *by nested PCR combined with agaros gel electrophoresis and ethidium-bromide staining, as previously described [5]. 

### 2.2. Determination of Buoyant Densities

Media of various densities were prepared from two colloidal density gradient media stock solutions; BactXtractor-Low density (BX-L) with a density of 1.057 g/mL and BactXtractor-High density (BX-H) with a density of 1.309 g/mL (FertiPro N.V., Industriepark Noord 32, 8730 Beernem, Belgium). To obtain other calculated densities required, BX-H was diluted with physiologic saline (0.86 –0.90 % NaCl, pH 7.5). All experiments were performed at room temperature.

To estimate the buoyant density of *L. intracellularis* the methodology described by Pertoft was used [16]. Briefly, 0.2 gram of a known PCR-positive faecal sample was mixed with 1.5 mL saline and incubated at room temperature for 10 minutes. One mL of the supernatant was mixed with 5.675 mL saline and 2.225 mL BX-H in a 15 mL tube. In a second tube, 6.625 mL saline and 2.225 mL BX-H were mixed with 50 *μ*L density marker beads (Amersham Biosciences AB, Uppsala, Sweden). The tubes were centrifuged in an ultracentrifuge with fixed angle rotor (Beckman Optima L80-XP, Beckman Coulter Inc., Fullerton, Calif, USA) at 15 000 × g for 30 minutes to create a self-generated continuous density gradient. Following centrifugation, the distance from the bottom of the second tube to the coloured bands created by the separated marker beads was measured and plotted against their densities. The volume per mm was calculated. From the first tube, aliquots of the sample were drawn from the bottom, and the volumes were measured and plotted in the density curve. Each aliquot was analysed by PCR. The procedure was repeated with two additional faecal samples known to be PCR positive for * L. intracellularis*. In a fourth tube, a sample previously shown to be strongly PCR inhibiting, as judged by the repeated failure to amplify the internal standard, was included to determine the buoyant density of major inhibitory components. The amount of *L. intracellularis*-specific DNA was estimated semiquantitatively by comparisons to an internal standard (mimic) [13].

### 2.3. PCR

PCR analysis for *L. intracellularis* was performed in accordance with Jacobson et al. [5] and 10^3^ internal controls (mimics) were included in each PCR reaction [13]. A positive control, originating from a tissue sample diagnosed as proliferative enteropathy at necropsy that was extracted by phenol-chloroform before PCR and containing ~10^7^ *L. intracellularis* organisms/mL, was included in all experiments. The forward primer sequence was 5′-TAT GGC TGT CAA ACA CTC CG-3′ and the reverse primer sequence was 5′-TGA AGG TAT TGG TAT TCT CC-3′ [17]. The reaction conditions consisted of 10 mM Tris-HCl (pH 8.3), 50 mM KCl, 2 mM MgCl_2_, 0.2 mM of each dNTP, and 1 U AmpliTaq Gold DNA polymerase (Applied Biosystems, Foster City, Calif, USA). All reactions were performed in a 25 *μ*L reaction volume. One *μ*L of the template was added and PCR was performed in a DNA thermal cycler (PTC-200, MJ Research Inc, Watertown, Mass, USA) starting with 10 minutes at 95°C followed by 35 cycles consisting of 95°C for 30 s, 55°C for 30 s, and 72°C for 30 s. When undiluted density media was included in the template, a prolonged annealing time (45 s) was used. All analyses were performed in duplicate or triplicate.

### 2.4. The Design of a Suitable Setup for Routine Analysis of Faecal Samples By Flotation

The discontinuous density gradient setup consisted of a high-density bottom phase, a middle phase of intermediate density, and a low-density top phase. Based on the estimated buoyant density of *L. intracellularis*, the densities were designed to float the bacterium from the bottom phase and concentrate the microbe to the interphase between the top and middle phases. Components of lower densities would be concentrated in the top phase while components of higher densities would be located in the middle and bottom phases. Small and water-soluble components such as free DNA and various PCR-inhibitors that were not affected by the applied g-force were expected to be located in the bottom phase. To maximize the separation of *L. intracellularis* from inhibitory factors, different densities of the middle and top phases were tested (Table 1). Approximately 0.5 g faecal sample was homogenized in 1 mL saline incubated for 10 minutes, and 1 mL supernatant was used as a sample applied in the bottom phase. In five tubes, a strongly positive sample was used and in one tube, a strongly inhibited sample was included. The two upper phases were carefully layered one by one to avoid disturbing the gradients. The samples were centrifuged in a swinging bucket rotor (#3047, Biofuge Stratos, Heraus Instruments GmbH, Hanau, Germany) at 5000 rpm (~4863 × g) or 30 minutes. From the upper interphase, samples (0.5–1 mL) were drawn with a sterile needle through the tube wall to avoid mixing the phases. Except for tube 1, the top, middle, and bottom phases were also collected for analysis. The samples were pelleted by centrifugation at 10000 rpm for 5 minutes and washed twice in saline, and the final pellet was resuspended in 0.2–0.3 mL saline and lysed at 95°C for 20 minutes prior to PCR. 

**Figure 1:**
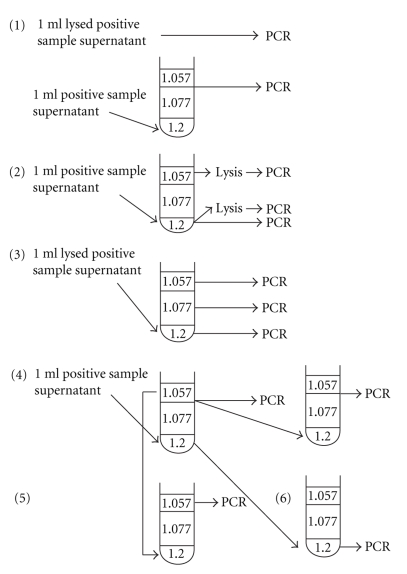
The design of a suitable setup for routine analysis of faecal samples by flotation. Six tubes were prepared and centrifuged in accordance with tube 4, Table 1 (tubes nos. 1 to 6). In tube 1, the sample was prepared by centrifugation and analyzed by PCR. An aliquot of the sample was lysed by boiling and analyzed by PCR without previous centrifugation. In tube 2, the top and the bottom phases were lysed before PCR. An aliquot of the bottom phase was analyzed by PCR without previous lysing. In tube 3, a lysed sample was included and all phases were analyzed by PCR. In tube 4, the upper interphase was subjected to a second centrifugation. Aliquots of the upper interphases from both centrifugations were subjected to PCR and the results were compared. The top and bottom fractions, respectively, from tube 4 were mixed with 1 mL supernatant from a negative faecal sample before centrifugation (tubes 5 and 6).

In addition, six tubes were prepared and centrifuged in accordance with tube 4, Table 1, to further analyse the overall distribution and recovery of *L. intracellularis* DNA in the gradient (Figure 1). To compare the amount of DNA recovered by flotation to the amount recovered by boiling [5, 18], tenfold dilutions of the sample prepared by either method were subjected to nested PCR together with 10^3^ mimics (Figure 1(1)). To determine if whole *L. intracellularis*-containing enterocytes were present, the top phase from tube 2 was analysed by PCR (undiluted and diluted 1:100) and likewise, the bottom phase was analysed to determine if free DNA was present. To confirm that free DNA would be allocated to the bottom phase only, a lysed sample was included in tube 3, the phases were collected separately, and undiluted samples and samples diluted 1:100 were subjected to PCR. To determine if *L. intracellularis* DNA was lost during the process, the interphase from tube 4 was collected and subjected to a second flotation. Subsequently, corresponding amounts of the interphases from the first and the second centrifugations were serially diluted and subjected to PCR. To determine if faecal particles would influence the recovery, 100 *μ*L sample from the bottom and top fractions, respectively (tube 4), was mixed with 1 mL supernatant from a negative faecal sample prior to centrifugation (tube 5 and 6). The samples (undiluted and diluted 1:100) were analysed by PCR. 

Further, to determine if the colloidal media would interfere with PCR, 1 *μ*L of *L. intracellularis* DNA precipitated by phenol/chloroform was subjected to PCR without being diluted, diluted 1:1 with saline, 1:1 with BX-L, or 1:1 with BX-H. 

### 2.5. The Design of a Small-Scale Setup

To adopt the procedure to standard laboratory equipment, a small-scale setup (bottom phase: 100 *μ*l sample mixed with 300 *μ*l BX-H, density 1.20 g/mL; middle phase: 304.1 *μ*L saline plus 95.9 *μ*L BX-H, density 1.074 g/mL; top phase: 315.8 *μ*L saline mixed with 84.2 *μ*L BX-H, density 1.065 g/mL) was tested on a panel of selected samples (Table 2). Six samples had previously been strongly positive for *L. intracellularis* when prepared by boiling and analysed by nested PCR, six samples had a weak positive reaction caused by a small amount of DNA in the sample as judged by comparison to the mimic [13], six had a weak positive reaction caused by partial inhibition of the PCR, and six samples had previously been completely inhibited. The samples were run in duplicate in a standard centrifuge with a fixed angle rotor (Hermle Z 160 M, Hermle Labortechnik GmbH, Wehingen, Germany) at 5 000 × g for 30 minutes. Following removal of 300 *μ*L of the top phase, the next 200 *μ*L sample was recovered from the upper interphase, lysed and centrifuged at 15 000 rpm for 30 s. before analysis by PCR. The results were compared to previous results (Table 2). 

### 2.6. Diagnosis of Clinical Samples

Further, 116 clinical samples originating from seven different farms were prepared by the optimized small-scale setup [19]. The bottom phase consisted of 150 *μ*L supernatant from the sample mixed with 450 *μ*l BX-H, the middle phase consisted of 450 *μ*L saline and 150 *μ*L BX-H with a density of 1.0772 g/mL, and the top phase was increased to 600 *μ*L BX-L. Following centrifugation at ~5000 × g for 30 minutes, the top phase was removed and the underlying 200–400 *μ*L was transferred to a new tube and lysed, and samples (undiluted and diluted 1:100) were analysed by PCR. Aliquots of the samples had previously also been prepared by a commercial kit (QIAamp DNA Stool Mini Kit, Qiagen Inc., Valencia, Calif, USA) and by boiling and these results were included for comparison (Table 3). 

## 3. Results

### 3.1. Determination of Buoyant Densities 

Following centrifugation, the self-generated continuous density gradient in the first tube yielded 25 fractions with volumes ranging from 155 to 510 *μ*l. Following PCR, all fractions in the density range of 1.138 to 1.051 g/mL were positive for *L. intracellularis*. Fractions with densities higher than 1.074 g/mL and lower than 1.065 g/mL contained less bacterial DNA (<10^5 ^target templates/mL) as determined semiquantitatively. In the repeated run on two additional positive samples, all fractions with densities >1.102 g/mL and <1.064 g/mL were negative and all fractions between these values were positive, each fraction containing ~10^2^–10^3^ DNA target templates. In the inhibited sample, weak inhibition was seen in four out of six fractions with densities between 1.102 and 1.074 g/mL, one fraction in this interval was completely inhibited, and one fraction was not inhibited. Fractions with densities around and slightly below 1.074 g/mL were not inhibited, but weak or complete inhibition was demonstrated in fractions with densities approaching 1.064 g/mL.

### 3.2. The Design of a Suitable Setup for Routine Diagnosis of Faecal Samples By Flotation

Following centrifugation, three phases were clearly visible. In the first setup, positive PCR reactions were demonstrated in all phases in tubes 1–4 (Table 1). As determined semiquantitatively, the bottom phases from tubes 2 and 3 contained less amount of DNA (~10^3^ target templates). In tube 5, the upper interphase was positive, the top phase was weakly positive, and the middle and bottom phases were negative. Hence, the buoyant density of *L. intracellularis* seems to be between 1.064 and 1.077 g/mL. In tube 6, the upper interphase and the top phase were inhibited whereas the middle and bottom phases were negative. Based on the results obtained, the selected setup consisted of a bottom phase with a density of 1.23 g/mL, a middle phase with a density of 1.077 g/mL, and a top phase with a density of 1.057 g/mL (tube 4, Table 1). 

In the comparison between the recoveries from samples prepared by buoyant density gradient centrifugation and from samples prepared by boiled lysate (Figure 1), both methods yielded positive PCR results in the undiluted sample and in samples diluted 1:10 and 1:100. However, a slightly stronger signal was achieved in samples prepared by boiling in the 1:100 dilution. In tube 2, a strong signal from the *L. intracellularis* amplicon was seen in the undiluted sample from the top phase. A positive result was also obtained from the undiluted bottom fraction, with a slightly stronger signal obtained in the lysed sample. All samples diluted 1:100 were negative. In tube 3, a positive result was achieved in all phases in both dilutions. The strongest amplicon signal was achieved in the top phase. In the undiluted samples, an unspecific band of ~1500 base pair was noted. Following the first centrifugation of the fourth tube, a weakly PCR-positive signal was achieved from the upper interphase diluted 1:100. Following a second centrifugation, the interphase diluted 1:100 was negative. The other fractions were negative. It was not possible to retrieve *Lawsonia* DNA following mixing with the negative sample and centrifugation (tube 5 and 6). 

PCR on the undiluted positive control or the control diluted with saline resulted in a single band of expected size (319 b p). When the control was mixed with BX-L, a weak unspecific band (~1500 b p) was seen and when the control was mixed with BX-H, a strong band of the same size was seen in addition to the positive control. 

### 3.3. The Design of a Small-Scale Setup

The results from the analyses of the panel of selected samples are shown in Table 2. The selected setup consisted of 150 *μ*l sample mixed with 450 *μ*l BX-H as bottom phase, 450 *μ*l saline mixed with 150 *μ*l BX-H as middle phase, and 300 *μ*l BX-L as top phase. 

### 3.4. Diagnosis of Clinical Samples

Of 24 samples previously lysed by boiling and judged as positive by nested PCR, 15 were judged as positive when prepared by the small-scale setup followed by conventional PCR. Of 17 samples previously judged as inhibited, two remained inhibited whereas 15 were judged as negative when prepared by the new method. In the previous experiment using a commercial kit for DNA extraction followed by PCR, 13 out of 24 samples were judged as positive and of the 17 previously inhibited samples, 16 were judged as negative and one as inhibited. All samples judged as negative by PCR on boiled lysate remained negative (Table 3). Utilising the preparation by boiling as “gold standard” and assuming that all inhibited samples are positive, the preparation by boiling would have a sensitivity of 70.7% and a specificity of 100%, the preparation by the commercial kit would have a sensitivity of 59.4% and a specificity of 98.7%, and the new method would have a sensitivity of 61.2% and a specificity of 97.4%. Utilising the preparation by boiling as “gold standard” and assuming that all inhibited samples are negative, the preparation by boiling would have a sensitivity of 100% and a specificity of 84.4%, the preparation by the commercial kit would have a sensitivity of 68.6% and a specificity of 98.9%, and the new method would have a sensitivity of 72.7 % and a specificity of 97.9%. 

## 4. Discussion

One of the major challenges in the diagnosis of *L. intracellularis* in faecal specimens is to recover a small number of pathogens in the bulk of faeces without the possibility to multiply the microbe by cultivation [10]. Further, amplification of microbial DNA by PCR may be prohibited by inhibitors present in faeces [11–13, 20–23]. Flotation can be used to concentrate all microbes within a defined density range at an easily identifiable position in the tube and separate them from soluble inhibitors and particles of other densities [1]. The technique is comparably fast and easy to perform. In the small scale design, standard laboratory equipment is used and the different phases seem to remain stable during collection of the interphase. The total time used for preparation is approximately the same as for the commercial kit (i.e. 1.5 hours/10 samples); however, excluding the time for centrifugation and incubation, the time used for manual preparation is about halved (i.e. 0.5 versus 1 hour/10 samples. Data not shown.) The method enables the demonstration of *L. intracellularis* DNA with a high specificity. In our study, however, the sensitivity seemed to be lower when compared to nested PCR performed on samples lysed by boiling. This may be explained by the fact that only the fraction containing a concentrate of whole *L. intracellularis* cells is analysed. It is also possible that some DNA is lost during preparation. The results are however still comparable to, or slightly better than, those obtained following DNA preparation by the commercial kit. Although both flotation and the kit combined with conventional PCR performed poorer as compared to boiling combined with nested PCR, the latter method is not suited for routine diagnosis [5, 24]. 

It is not clear why PCR-positive reactions for *L. intracellularis* were obtained from all phases in some samples. Theoretically, the time or the speed of centrifugation could be too short to allow the bacteria to pass through the higher density layers and concentrate in the upper interphase. However, positive PCR results for *L. intracellularis* were also achieved from the top phase. Further, incautious handling of the tubes might cause stirring of the phases. However, the different phases were clearly visible both before and after centrifugation. In addition, collection of the sample through the tube wall did not improve the results. This phenomenon has not been described in similar setups with pure cultures of bacteria of similar density [1]. A third explanation could be that *L. intracellularis* may be shed both within sloughed epithelial cells, as free organisms and as bacterial DNA from disintegrated bacteria, thereby obtaining a range of densities. Further, it is not known if *L. intracellularis*is extruded from the intestines at a particular growth phase. Longitudinal studies on *C. jejuni* have shown that the buoyant densities changed over a range of 1.076 to 1.130 g/mL, indicating that the density may alter depending on the growth phase [1]. Hence, free DNA originating from lysed bacteria would be located in the bottom phase [1], whereas bacteria in exfoliated enterocytes shed in faeces [25] would be retained in the top phase. In fact, this has proven to be a possible way to separate dead and live bacteria [1], which would be an advantage, for instance, in the validation of treatment regimens. In the continuous gradient, all fractions from one sample were PCR positive for *L. intracellularis* although less DNA was demonstrated at higher and lower densities. However, the density marker beads simultaneously ran in a separate tube and under similar conditions created distinct, coloured bands at predicted locations. Hence, it seems that in clinical specimens, *L. intracellularis *is not of homogenous density. It would have been preferable to test this hypothesis by performing the studies on pure culture of bacteria; however, due to the difficulties to cultivate *L. intracellularis*, this was not possible. 

It has been speculated that faeces contain several inhibitors of different composition [11, 12, 26–29]. This was also indicated in the present study, where PCR detection of *L. intracellularis* was slightly or completely inhibited in three fractions with densities between 1.102 and 1.074 and in two fractions with densities just below 1.064 g/mL. Further, we have previously demonstrated the presence of inhibitory factors in the protein, lipid, fibre and mineral fraction of faeces (data not shown). This may explain the difficulties described in several studies to overcome the PCR inhibition in faecal samples, since most methods are designed to circumvent one single inhibitory mechanism [5, 27]. The results in the present study may also indicate that some inhibitory substances have a density close to that of *L. intracellularis*. 

The nature of the unspecific band of approximately 1500 bp seen following electrophoresis and ethidium bromide staining of the colloidal media has not been elucidated. Ethidium bromide specifically stains DNA, but attempts to purify and identify the product have been unsuccessful (data not shown). However, it does not seem to interfere with PCR or the detection of *L. intracellularis*.

Flotation concentrates bacteria of desired density at a certain location. Theoretically it may therefore have a potential for purification and retention of *L. intracellularis,* for instance, prior to cultivation. This may be a desirable property, since purification of *L. intracellularis* traditionally is based on filtering through sequentially smaller membranes, which is time consuming and requires special equipment [30].

In opposite to Percoll, the present technique is based on silanised silicon particles resulting in a clear solution. Hence, the media do not interfere with fluorescence-based methods such as real-time PCR [1]. As compared to Percoll, the particles are also more stable. However, when used without further washing, convection in the hot solution is slower and therefore the annealing time in the PCR reaction might need to be adjusted. In addition, caution should be paid to samples with high lipid content that may disrupt the gradients.

 In conclusion, the performance of the buoyant density gradient centrifugation combined with conventional PCR is comparable to or slightly better than the method presently used for routine diagnosis of *L. intracellularis* in faecal samples, that is, preparation by a commercial kit combined with conventional PCR. In our hands, the new method is also faster and easier to perform and can be used to separate the microbe from soluble inhibitors and particles of other densities. In addition, it may be used to purify the microbe prior to cultivation. Based on the results obtained in this study, the buoyant density of *L. intracellularis* seems to be between 1.064 and 1.077 g/mL.

## Figures and Tables

**Table 1:** The design of six different density gradient setups to establish a suitable flotation setup for routine analysis of faecal samples. The supernatant from a PCR-positive (pos) or inhibiting (inh) faecal sample was mixed with a high density medium (BX-H) and over layered by two phases of decreasingly lower densities (BX-H mixed with saline, BX-L, or BX-L mixed with saline). Five different density gradient setups were tested (tube 1-5) and a previously PCR-inhibiting sample was included in tube 6 using the same density setup as tube 4.

	Tube	1	2	3	4	5	6
Top phase	Density (g/mL)	1.057	1.057	1.057	1.057	1.028	1.057
	BX-L (mL)	2.0	2.0	2.0	2.0	1.0	2.0
	Saline (mL)	—	—	—	—	1.0	—
Middle phase	Density (g/mL)	1.1543	1.1286	1.1029	1.0772	1.057	1.0772
	BX-H (mL)	3.0	2.5	2.0	1.5	—	1.5
	Saline (mL)	3.0	3.5	4.0	4.5	—	4.5
	BX-L (mL)	—	—	—	—	6	—
Bottom phase	Density (g/mL)	1.23	1.23	1.23	1.23	1.23	1.23
	BX-H (mL)	3.0	3.0	3.0	3.0	3.0	3.0
	Sample (mL)	1.0 (pos)	1.0 (pos)	1.0 (pos)	1.0 (pos)	1.0 (pos)	1.0 (inh)

**Table 2:** The results from PCR analyses of 24 selected samples prepared by an optimized small-scale flotation setup. Aliquots of the samples had previously been judged as strongly positive^a^, weakly positive^b^, partly inhibited^c^ (inh), or inhibited^d^ by boiling lysate combined with nested PCR.

Samples, previous results				
Results, flotation	Strongly positive, n = 6	Weakly positive, not inh, n = 6	Weakly positive, partly inh, n = 6	Inhibited, n = 6
Positive	4	1	4	—
Negative	2	5	2	5
Inhibited	—	—	—	1

^a^The signal from the sample amplicon was equal to the positive control and the internal control was outnumbered.

^b^The signal from the sample amplicon was weaker than the signal from the internal control.

^c^The signal from the sample amplicon was equal to the signal from the sample^b^ but the internal control was not visualised.

^d^No signal was visualised.

**Table 3:** The results from PCR analysis of 116 clinical samples previously judged as positive, negative, or inhibited by boiling and nested PCR, prepared by the finally selected flotation setup. The results from two other, previously used methods are included for comparison.

Sample preparation method		Results	
	Positive	Inhibited	Negative
Boiled lysate + nested PCR	24	17	75
QIAamp DNA Stool Mini Kit + PCR	13	1	102
Flotation + PCR	15	2	99

## References

[1] Wolffs PFG, Glencross K, Norling B, Griffiths MW (2007). Simultaneous quantification of pathogenic *Campylobacter* and *Salmonella* in chicken rinse fluid by a flotation and real-time multiplex PCR procedure. *International Journal of Food Microbiology*.

[2] Stege H, Jensen TK, Møller K, Bækbo P, Jorsal SE (2000). Prevalence of intestinal pathogens in Danish finishing pig herds. *Preventive Veterinary Medicine*.

[3] Cooper DM, Gebhart CJ (1998). Comparative aspects of proliferative enteritis. *Journal of the American Veterinary Medical Association*.

[4] Zhang P, Gebhart CJ, Burden D, Duhamel GE (2000). Improved diagnosis of porcine proliferative enteropathy caused by *Lawsonia intracellularis* using polymerase chain reaction-enzyme-linked oligosorbent assay (PCR-ELOSA). *Molecular and Cellular Probes*.

[5] Jacobson M, Aspan A, Heldtander Königsson M (2004). Routine diagnostics of *Lawsonia intracellularis* performed by PCR, serological and post mortem examination, with special emphasis on sample preparation methods for PCR. *Veterinary Microbiology*.

[6] Guedes RMC, Gebhart CJ, Winkelman NL, Mackie-Nuss RAC, Marsteller TA, Deen J (2002). Comparison of different methods for diagnosis of porcine proliferative enteropathy. *Canadian Journal of Veterinary Research*.

[7] Wattanaphansak S, Asawakarn T, Gebhart CJ, Deen J (2008). Development and validation of an enzyme-linked immunosorbent assay for the diagnosis of porcine proliferative enteropathy. *Journal of Veterinary Diagnostic Investigation*.

[8] Jordan DM, Knittel JP, Roof MB, Schwartz K, Larson D, Hoffman LJ (1999). Detection of *Lawsonia intracellularis* in swine using polymerase chain reaction methodology. *Journal of Veterinary Diagnostic Investigation*.

[9] Kroll JJ, Eichmeyer MA, Schaeffer ML, McOrist S, Harris DL, Roof MB (2005). Lipopolysaccharide-based enzyme-linked immunosorbent assay for experimental use in detection of antibodies to *Lawsonia intracellularis* in pigs. *Clinical and Diagnostic Laboratory Immunology*.

[10] Dittmar M, Hoelzle LE, Hoelzle K (2003). Diagnosis of porcine proliferative enteropathy: detection of *Lawsonia intracellularis* by pathological examinations, polymerase chain reaction and cell culture inoculation. *Journal of Veterinary Medicine Series B*.

[11] Lantz P-G, Abu Al-Soud  W, Knutsson R, Hahn-Hägerdal B, Rådström P (2000). Biotechnical use of polymerase chain reaction for microbiological analysis of biological samples. *Biotechnology Annual Review*.

[12] Wilson IG (1997). Inhibition and facilitation of nucleic acid amplification. *Applied and Environmental Microbiology*.

[13] Jacobson M, Englund S, Ballagi-Pordány A (2003). The use of a mimic to detect polymerase chain reaction-Inhibitory factors in feces examined for the presence of *Lawsonia intracellularis*. *Journal of Veterinary Diagnostic Investigation*.

[14] Uyttendaele M, Debevere J, Lindqvist R (1999). Evaluation of buoyant density centrifugation as a sample preparation method for 
NASBA-ELGA detection of *Campylobacter jejuni* in foods. *Food Microbiology*.

[15] Thisted Lambertz S, Lindqvist R, Ballagi-Pordány A, Danielsson-Tham M-L (2000). A combined culture and PCR method for detection of pathogenic *Yersinia enterocolitica* in food. *International Journal of Food Microbiology*.

[16] Pertoft H (2000). Fractionation of cells and subcellular particles with Percoll. *Journal of Biochemical and Biophysical Methods*.

[17] Jones GF, Ward GE, Murtaugh MP, Lin G, Gebhart CJ (1993). Enhanced detection of intracellular organism of swine proliferative enteritis, ileal symbiont intracellularis, in feces by polymerase chain reaction. *Journal of Clinical Microbiology*.

[18] Møller K, Jensen TK, Jorsal SE, Leser TD, Carstensen B (1998). Detection of *Lawsonia intracellularis*, *Serpulina hyodysenteriae*, weakly 
beta-haemolytic intestinal spirochaetes, *Salmonella enterica*, and haemolytic *Escherichia coli* from swine herds with and without diarrhoea among growing pigs. *Veterinary Microbiology*.

[19] Jacobson M, Wennerbo S, Aspan A, Gunnarsson A, Fellström C The importance of faecal sampling techniques in the PCR diagnosis of *Lawsonia intracellularis*.

[20] Altwegg M (1995). General problems associated with diagnostic applications of amplification methods. *Journal of Microbiological Methods*.

[21] McOrist AL, Jackson M, Bird AR (2002). A comparison of five methods for extraction of bacterial DNA from human faecal samples. *Journal of Microbiological Methods*.

[22] Lawson GHK, Gebhart CJ (2000). Proliferative enteropathy. *Journal of Comparative Pathology*.

[23] Gebhart  C *Lawsonia intracellularis* infections.

[24] Neumaier M, Braun A, Wagener C (1998). Fundamentals of quality assessment of molecular amplification methods in clinical diagnostics. *Clinical Chemistry*.

[25] McOrist S, Roberts L, Jasni S (1996). Developed and resolving lesions in porcine proliferative enteropathy: possible pathogenetic mechanisms. *Journal of Comparative Pathology*.

[26] Widjojoatmodjo MN, Fluit AC, Torensma R, Verdonk GPHT, Verhoef J (1992). The magnetic immuno polymerase chain reaction assay for direct detection of Salmonellae in fecal samples. *Journal of Clinical Microbiology*.

[27] Cavallini A, Notarnicola M, Berloco P, Lippolis A, Di Leo A (2000). Use of a macroporous polypropylene filter to allow identification of bacteria by PCR in human fecal samples. *Journal of Microbiological Methods*.

[28] Monteiro L, Bonnemaison D, Vekris A (1997). Complex polysaccharides as PCR inhibitors in feces: helicobacter pylori model. *Journal of Clinical Microbiology*.

[29] Lou Q, Chong SK, Fitzgerald JF, Siders JA, Allen SD, Lee C-H (1997). Rapid and effective method for preparation of fecal specimens for PCR assays. *Journal of Clinical Microbiology*.

[30] Lawson GHK, McOrist S, Jasni S, Mackie RA (1993). Intracellular bacteria of porcine proliferative enteropathy: cultivation and maintenance in vitro. *Journal of Clinical Microbiology*.

